# Type I Collagen Suspension Induces Neocollagenesis and Myodifferentiation in Fibroblasts *In Vitro*

**DOI:** 10.1155/2020/6093974

**Published:** 2020-06-26

**Authors:** Francesca Lombardi, Paola Palumbo, Francesca Rosaria Augello, Ilaria Giusti, Vincenza Dolo, Luca Guerrini, Maria Grazia Cifone, Maurizio Giuliani, Benedetta Cinque

**Affiliations:** ^1^Department of Life, Health & Environmental Sciences, University of L'Aquila, L'Aquila 67100, Italy; ^2^Unit of Plastic and Reconstructive Surgery, Casa di Cura “Di Lorenzo” SrL, Via Vittorio Veneto 37, Avezzano, 67051 L'Aquila, Italy

## Abstract

The ability of a collagen-based matrix to support cell proliferation, migration, and infiltration has been reported; however, the direct effect of an aqueous collagen suspension on cell cultures has not been studied yet. In this work, the effects of a high-concentration aqueous suspension of a micronized type I equine collagen (EC-I) have been evaluated on a normal mouse fibroblast cell line. Immunofluorescence analysis showed the ability of EC-I to induce a significant increase of type I and III collagen levels, parallel with overexpression of crucial proteins in collagen biosynthesis, maturation, and secretion, prolyl 4-hydroxylase (P4H) and heat shock protein 47 (HSP47), as demonstrated by western blot experiments. The treatment led, also, to an increase of *α*-smooth muscle actin (*α*-SMA) expression, evaluated through western blot analysis, and cytoskeletal reorganization, as assessed by phalloidin staining. Moreover, scanning electron microscopy analysis highlighted the appearance of plasma membrane extensions and blebbing of extracellular vesicles. Altogether, these results strongly suggest that an aqueous collagen type I suspension is able to induce fibroblast myodifferentiation. Moreover, our findings also support in vitro models as a useful tool to evaluate the effects of a collagen suspension and understand the molecular signaling pathways possibly involved in the effects observed following collagen treatment *in vivo*.

## 1. Introduction

Collagen is the most abundant protein in the human body, where it is responsible for structure, stability, and strength, especially within the dermal layers [1]. Alterations in the structure and quantity of collagen fibers are the main changes observed in aged skin [2, 3]. In fact, while in young skin collagen fibrils are intact, abundant, tightly packed, and well-organized, with advancing age, they undergo progressive loss and fragmentation, causing the atrophy and collapse of skin tissue and resulting in skin wrinkling and thinning, loss of elasticity, drying, and roughing [4–6]. An increase of collagen degradation together with the reduction of its biosynthesis is implicated in altered collagen homeostasis, which results in a net collagen deficiency [7–9]. Previous studies show that skin fibroblasts isolated from older people have altered migration, premature senescence, reduced proliferative response, and defects in matrix generation compared with dermal fibroblasts of young people [10–13]. In youthful skin, fibroblasts attach to the surrounding intact extracellular matrix (ECM). This attachment allows fibroblasts to exert mechanical forces on the surrounding ECM and to spread and maintain a standard elongated shape [2]. In aged skin, the progressive ECM degradation led to fibroblast size decrease, reduced elongation, and collapsed morphology resulting in an impairment of fibroblast adhesion [14–16]. Of note, aged fibroblasts resist myofibroblast differentiation [12]. Age, together with diabetes and vasculopathy, is the major risk factor associated with the development of chronic wounds, which are often characterized by high ECM degradation [17, 18]. The highly inflammatory, ischemic, and hypoxic environment of chronic wounds was shown to impair fibroblast proliferation, myofibroblast differentiation, and collagen ECM formation [19]. Moreover, reduced granulation tissue contraction caused by impaired myofibroblast and reduced reepithelialization further delay wound closure [20, 21]. Since myofibroblasts play crucial roles in preserving skin homeostasis and regulating skin repair and regeneration [22], stimulating myofibroblast formation and survival could be a promising potential treatment option for chronic wounds [23].

Collagen has been recognized for its biological action, with low immunogenicity and great potential in the pharmaceutical and clinical fields [24–26]. The most abundant collagens in human skin are types I and III, which form the structural fibrils and are responsible for the strength and stiffness of the tissue [27]. Type I collagen, the most used for medical applications, is considered the reference standard due to its high biocompatibility with the human body [26, 28]. The beneficial effects of topical administration and oral consumption of collagen on a variety of dermal parameters have been reported by a plethora of *in vivo* studies [29–32]. The ability of a collagen-based matrix to support cell proliferation, migration, and infiltration has also been studied *in vitro* for the development of collagen scaffolds for skin tissue regeneration [33, 34]. Collagen can be used alone or in combination with other molecules with therapeutic potential in wound treatment, such as nitric oxide, one of the most important players in the regulation of the wound repair [35]. In this regard, a nanofibrous scaffold containing collagen type I peptides and a NO donor was shown to enhance the spreading of F-actin and the proliferation of fibroblast cells *in vitro* [36], suggesting a new perspective for the treatment of nonhealing wounds. In addition, the concurrent use of probiotic bacteria and collagen hydrogel-scaffold dressing improved burn wound healing in rat [37], indicating the potential ability of a collagen-based scaffold to support and enhance the effects of probiotics, known to have beneficial and prohealing propriety on the skin [38–41].

Despite a plethora of studies investigating the effect of collagen scaffold on *in vitro* models, according to our knowledge, no study is available on the direct effect of collagen suspension on cell cultures. Thus, the purpose of the present work was to investigate the effects of high-concentration collagen suspension on fibroblasts *in vitro* to evaluate its ability to stimulate the production of new collagen and myodifferentiation. To this aim, we use a treatment based on micronized type I equine collagen aqueous suspension (EC-I). The micronization is a process that reduces the size of protein aggregates improving their rate of dissolution and preserves the structural integrity of the protein [42, 43]. The influence of EC-1 was analyzed on cell viability and expression of collagen I and III as well as P4H and HSP47, two key proteins involved in collagen synthesis [44–46]. Moreover, the EC-1-induced myodifferentiation was evaluated analyzing the ability of EC-1 to promote cytoskeletal reorganization, expression of *α*-SMA (the most used myofibroblast marker), and cell morphology modifications through scanning electron microscopy (SEM).

## 2. Materials and Methods

### 2.1. NIH 3T3 Cell Line and Treatments

Swiss NIH 3T3 mouse embryo fibroblast cell line was purchased from American Type Culture Collection (ATCC, Manassas, VA, USA). NIH 3T3 cells were cultured in complete medium, i.e., DMEM high glucose (Dulbecco's Modified Eagle Medium, EuroClone, West York, UK) supplemented with 10% (*v*/*v*) bovine calf serum (BCS), 2 mM L-glutamine, 100 U/ml penicillin, and 100 *μ*g/ml streptomycin (EuroClone, West York, UK). Culture conditions were kept constant at 37°C in a 5% CO_2_ humidified atmosphere. After reaching 80% confluence, the cells were harvested using a trypsin-EDTA solution to detach them from the bottom of the flask and seeded, as below specified, into a sterile tissue culture 6-well plate, 12-well plate, or onto coverslips (Becton Dickinson, San Jose, CA). Micronized type I equine collagen (LINeRASE, 100 mg of type I equine collagen powder, kindly provided by Taumedika srl, Rome, Italy) was suspended in 5 ml of complete medium (concentration 20 mg/ml) and used at the final concentrations of 0.4, 4, or 8 mg/ml.

### 2.2. Cell Viability Assay and Apoptosis Analysis

NIH 3T3 cells were seeded into 6-well plates (18,000 cells/cm^2^), grown overnight, and incubated with EC-I suspension at 0.4, 4, or 8 mg/ml up to 48 h in complete medium. The cells were then harvested using a trypsin solution, centrifuged for 10 min at 400 × g, and the resuspended pellets were incubated with a 0.04% Trypan blue (EuroClone, West York, UK) solution for 5 min to analyze cell number and viability. Nontreated cells (control) were also analyzed and served as negative controls. Cells were transferred to a Bürker counting chamber and counted by microscopy (Eclipse 50i, Nikon Corporation, Japan). The cell numbers and the percentage of live and dead cells were registered. Treatment with the detergent Triton X (0.1%) resulted in a significant reduction (*P* ≤ 0.01) in the cell viability (positive control). The apoptosis analysis was carried out by cell DNA staining with propidium iodide (PI). NIH 3T3 grown in the 6-well plates (seeded at 18,000 cells/cm^2^), untreated or treated with EC-I at 0.4, 4, and 8 mg/ml for 24 h, were detached with trypsin solution, centrifuged at 310 × g for 10 min at 4°C, counted, and fixed in ice-cold ethanol (70%) at 4°C for 30 min. The fixed cells were transferred to plastic BD tubes (Becton Dickinson, San José, CA, USA), washed twice with the ice-cold PBS, and stained with a mixture solution of PI (50 *μ*g/ml), Nonidet-P40 (0.1% *v*/*v*), and RNase A (6 *μ*g/10^6^ cells) (all from Sigma-Aldrich, Saint Louis, MO, USA) for 1 h in the dark at 4°C. The apoptotic cells were determined by their hypochromic subdiploid nuclei staining profiles using FACSCalibur flow cytometry and Cell Quest software program (BD Instruments Inc., San José, CA, USA).

### 2.3. Total Collagen Quantification

The collagen content in NIH 3T3 cells was evaluated using Sirius Red/Fast Green Collagen Staining Kit (Chondrex Inc., Redmond, WA, USA), a semiquantitative assay kit to determine the amounts of collagen and noncollagenous proteins in cultured cells, according to the manufacturer's instruction. Briefly, NIH 3T3 cells grown on a 12-well plate (seeded at 18,000 cells/cm^2^) were treated with EC-I suspension at 0.4, 4, or 8 mg/ml for 24 h in complete medium; then, the wells were washed with PBS and 1 ml of Kahle fixative (60 ml distilled water, 28 ml 96% ethanol, 10 ml 37% formaldehyde, and 2 ml glacial acetic acid) was added and the plate was incubated for 10 minutes at room temperature. 0.6 ml of dye solution was added, and after 30 minutes at room temperature, the dye solution was aspirated, and the wells were rinsed with 1 ml of distilled water repeatedly until the water ran clear. 1 ml of Dye Extraction Buffer was added on each sample and gently mixed by pipetting until the colour was eluted. The eluted dye solution was collected, and the OD values were read at 540 nm and 605 nm with a spectrophotometer (Bio-Rad, Hercules, California, USA). To calculate the amount of collagen, the OD 540 value was corrected by subtracting the contribution of Fast Green at 540 nm, which is 29.1% of the OD 605 value. The colour equivalence (OD values/*μ*g protein) is 0.0378 for collagen and 0.00204 for noncollagenous proteins at OD 540 and 605, respectively [47]. Noncollagenous protein values were used to normalize the results of samples.

### 2.4. Western Blot Analysis

For western blot analyses, cells seeded into 6-well plates (18,000 cells/cm^2^), grown overnight and then untreated or treated with 4 mg/ml EC-I suspension in complete medium for 24 h, were harvested, washed in PBS, and lysed in RIPA buffer (Merck KGaA, Darmstadt, Germany) containing 100 mM protease inhibitor cocktail (Sigma-Aldrich, St. Louis, MO, USA). The samples were assayed for protein content with DC Protein Assay (Bio-Rad, Hercules, CA) using BSA as standard. 25 *μ*g of proteins was mixed with sample buffer, boiled for 5 min at 100°C, and separated by 12.5% SDS-polyacrylamide gel electrophoresis. Proteins were transferred onto 0.45 *μ*m nitrocellulose membrane sheets (Bio-Rad, Hercules, CA) for 1 h at 4°C at 70 V using a Mini Trans-Blot Cell apparatus (Bio-Rad, Hercules, CA). Membranes were blocked with 5% nonfat dry milk for 1 h at room temperature and then incubated overnight at 4°C with rabbit monoclonal antibody anti-heat shock protein 47 (HSP47) 1 : 1,000, goat polyclonal antibody anti-prolyl 4-hydroxylase subunit alpha-1 (P4HA1) 1 : 1,000, rabbit monoclonal antibody anti-*α*-actin smooth muscle (ACTA2, *α*-SMA) 1 : 1,000 (OriGene, Rockville, Maryland, USA), or with goat polyclonal antibody anti-*β*-actin 1 : 1,000 (Santa Cruz Biotechnology, Inc., Dallas, TX, USA.). Horseradish peroxidase- (HRP-) conjugated goat anti-rabbit IgG secondary antibody 1 : 4,000 was used for anti-HSP47 and anti-*α*-SMA antibodies and horseradish peroxidase- (HRP-) conjugated rabbit anti-goat IgG secondary antibody 1 : 4,000 for anti-P4HA1 and anti-*β*-actin antibodies (Millipore EMD, Darmstadt, Germany). Immunoreactive bands were visualized by enhanced chemiluminescence (ECL, Amersham Pharmacia Biotech), according to the manufacturer's instructions. Band relative densities were determined using a chemiluminescence documentation system ALLIANCE (UVITEC, Cambridge UK), and values were given as relative units. Immunoblot data were normalized to *β*-actin protein levels.

### 2.5. Immunofluorescence Staining

NIH 3T3 cells grown on coverslips in a 12-well plate (seeded at 5,000 cells/cm^2^) were treated with or without EC-I suspension at 4 mg/ml for the indicated time points. The coverslips were then washed with PBS, fixed with 4% formaldehyde for 20 min, permeabilized with 0.1% Triton X-100 (Sigma-Aldrich, St. Louis, MO, USA) for 5 min, and blocked with 3% BSA (Sigma-Aldrich) for 20 min at room temperature. Subsequently, cells were incubated overnight at 4°C with rabbit polyclonal antibody anti-COL1A1 (Boster Biological Technology, Pleasanton, CA, USA) 1 : 250, rabbit polyclonal antibody anti-COL3A1 (Elabscience, Houston, Texas, USA) 1 : 250, or rabbit monoclonal antibody anti-*α*-actin (smooth muscle) (*α*-SMA, OriGene, Rockville, Maryland, USA) 1 : 250. Then, the coverslips were stained using a FITC conjugated goat anti-rabbit polyclonal IgG secondary antibody (Millipore EMD, Darmstadt, Germany) 1 : 1,000 for 1 h at room temperature, washed and incubated with TRITC labeled phalloidin (Sigma-Aldrich) for 45 min at room temperature. The coverslips were mounted with VECTASHIELD® Antifade Mounting Medium with DAPI (Vector Laboratories, Inc., Burlingame, CA, USA) and examined at ×100 magnifications with a fluorescent microscope (Eclipse 50i, Nikon, Tokyo, Japan).

### 2.6. Scanning Electron Microscopy

**Figure 1:**
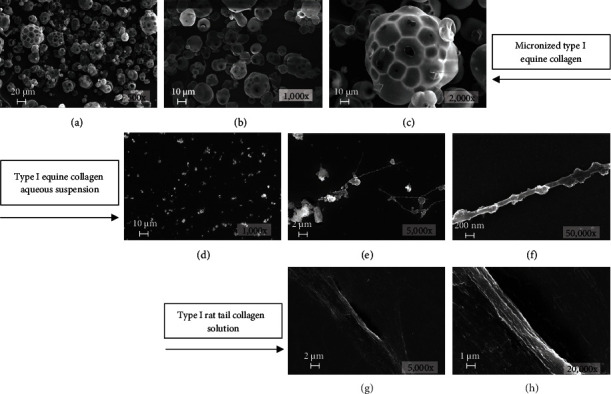
Scanning electron micrograph images of micronized EC-I ((a) 500x, (b) 1000x, and (c) 2000x magnification, respectively), EC-I aqueous solution ((d) 1000x, (e) 5000x, and (f) 50,000x magnification, respectively), and type I rat tail collagen solution ((g) 5000x and (h) 20,000x magnification, respectively).

Scanning electron microscopy (SEM) was carried out on NIH 3T3 cells grown on coverslips in a 12-well plate (seeded at 5,000 cells/cm^2^), untreated or treated with 4 mg/ml EC-I for 4 or 24 h, on water-dissolved EC-I (4 mg/ml) adhered on glass coverslips for 15 min, and on type I rat tail collagen solution 4 mg/ml (SERVA Electrophoresis GmbH, Heidelberg, Germany) let to adhere on glass coverslips overnight at 4°C. The coverslips were then fixed with 2% glutaraldehyde (Electron Microscopy Sciences, Hatfield, PA, USA) in PBS for 10 (for NIH/3T3 cells) or 15 min (for water-dissolved EC-I and type I rat tail collagen solution), briefly rinsed with PBS and water and then dehydrated in ethanol solutions 30–50–70–90% in H_2_O and three times 100%, for 10 min each. For HMDS drying, the samples were immersed for 3 min in 100% HMDS (Electron Microscopy Sciences, Hatfield, PA, USA), and then, the excess HMDS was blotted away by filter paper. The samples were then transferred to a desiccator for 25 min to avoid contamination with water; the samples were mounted on stubs, sputter-coated with chromium in a Quorumtech Q 150T ES Turbo chromium sputter, and detected via a Zeiss Gemini SEM 500. SEM was also performed on not dissolved EC-I scattered on adhesive carbon conductive tabs (PELCO Tabs™, Ted Pella Inc., Redding, CA, USA) mounted on stubs, sputter-coated, and detected as described above. SEM images of EC-I powder, water-dissolved EC-I, and type I rat tail collagen solution are shown in Figure 1.

### 2.7. Statistical Analysis

Data were analyzed using Prism 6.0 (GraphPad Software, San Diego, CA, USA). Results are expressed as the mean ± SD of one experiment performed in duplicate or mean ± SEM of three independent experiments performed in duplicate. For comparison between two means, Student's unpaired *t*-test was used. For comparisons of the mean values among groups, a one-way ANOVA followed by Dunnett *post hoc* test or a two-way ANOVA followed by Bonferroni *post hoc* test was used. Values of *P* less than 0.05 were accepted as significant.

## 3. Results

### 3.1. Effects of EC-I on NIH 3T3 Cell Viability and Collagen Level

**Figure 2:**
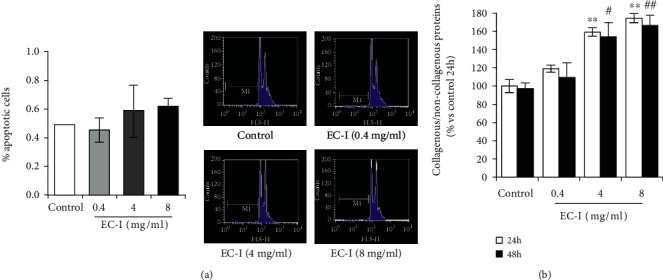
Effect of EC-I treatment on NIH 3T3 cell apoptosis and collagen levels. (a) Data from cytofluorimetric analyses of apoptosis after treatment for 24 h with 0.4, 4, or 8 mg/ml EC-I, expressed as mean%apoptotic cells ± SD. The results are from one representative out of three independent experiments in duplicate. The representative flow cytometry profiles are also shown. (b) Collagen content in fibroblasts untreated (control) or treated with 0.4, 4, or 8 mg/ml of EC-I suspension for 24 or 48 h evaluated using Sirius Red/Fast Green Collagen Staining Kit. The ratio of collagenous protein concentration/noncollagenous protein concentration reported in the histograms is expressed as mean ± SD and is from one representative out of three experiments in duplicate. ∗∗*P* < 0.01*vs*. control at 24 h; #*P* < 0.05 and *^##^P* < 0.01*vs*. control at 48 h.

**Figure 3:**
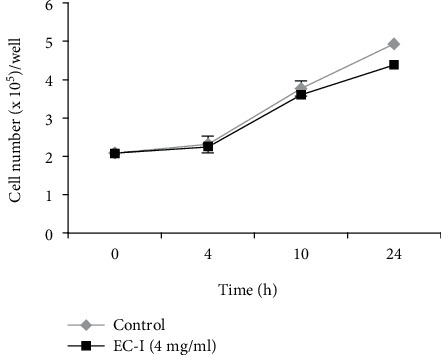
Effect of EC-I at 4 mg/ml on cell proliferation rate up to 24 h. The values represent the cell number expressed as mean ± SD from one representative out of three experiments in duplicate.

NIH 3T3 fibroblasts were incubated for 24 or 48 h with EC-I suspension at 0.4, 4, and 8 mg/ml (concentration range commonly used for collagen scaffold preparation [48–51]), after which the cell number and viability were analyzed. The results of the cell count with Trypan blue dye exclusion test showed that exposure of fibroblasts up to 48 h to all three concentrations of EC-I suspension did not influence cell number and viability (ever >95-98%). Flow cytometry analysis of hypochromic subdiploid PI staining showed no difference in the apoptosis level between control and EC-I-treated cells. The results from a representative experiment out of three independent experiments in duplicate are shown in Figure 2. Next, we investigated the effects of EC-I on the intracellular collagen level using a dye combination of Sirius Red and Fast Green, able to distinguish collagenous from noncollagenous proteins. The spectrophotometric determination of collagenous proteins normalized versus noncollagenous protein concentration showed that, while 0.4 mg/ml EC-I did not influence the collagen content with respect to untreated cells, both 4 and 8 mg/ml induced a significant increase of collagen level either at 24 or 48 h. Data expressed as mean ± SD of a representative experiment in duplicate from three independent experiments are shown in Figure 2(b). Since 0.4 mg/ml did not affect collagen production and the treatment with 4 and 8 mg/ml gave comparable results, the concentration of 4 mg/ml was used for the following experiments. EC-I treatment at 4 mg/ml had no effect on cell proliferation analyzed up to 24 h (Figure 3).

### 3.2. Effect of EC-I on Type I and III Collagen Synthesis

**Figure 4:**
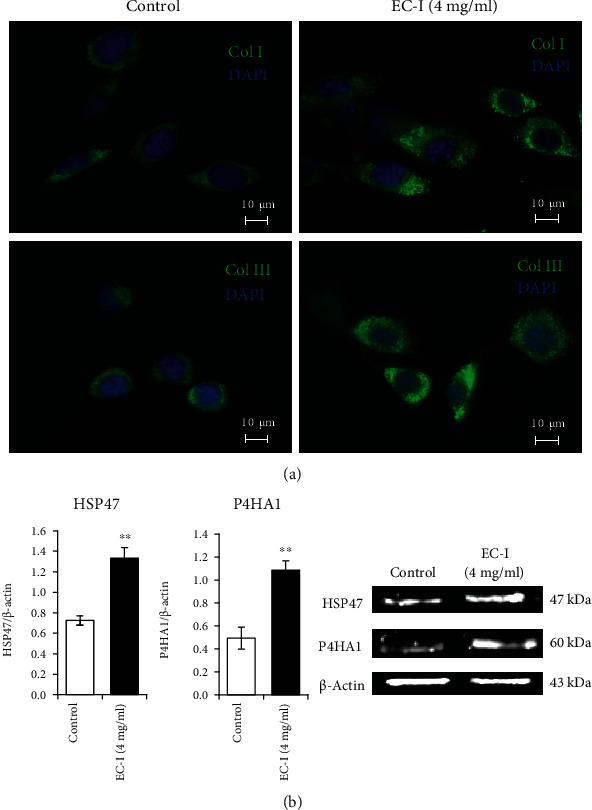
Effect of EC-I treatment on collagen type I and III and HSP47/P4HA1 expression. (a) Representative immunofluorescence images of NIH 3T3 cells treated for 24 h with EC-I suspension (4 mg/ml) and stained with antibodies against collagen type I or type III (green). Nuclei were counterstained with DAPI (blue) (magnification 100x). The results are representative of three independent experiments in duplicate. (b) Immunoblotting assay for HSP47 and P4HA1. Following densitometric analysis, obtained values were normalized *vs*. *β*-actin. Data are from three independent experiments in duplicate, and values are expressed as mean ± SEM. ∗∗*P* < 0.01*vs*. control. A representative immunoblot of HSP47 and P4HA1 is also shown.

The effects of EC-I on type I and III collagen production were evaluated by immunofluorescence. After 24 h of incubation with or without EC-I at 4 mg/ml, the cells were stained for collagen type I (Col Ia1, green) and III (Col IIIa1, green) and for nuclei (DAPI, blue). As shown in the representative images reported in Figure 4(a), the cells treated with EC-I showed more intense and widespread collagen type I (Col Ia1) and III (Col IIIa1) staining compared to untreated cells, thus suggesting an increase of collagen synthesis. Of note, since all the cells of the coverslips show 100% positivity for both collagen I and III staining, it is fair to conclude that the same cell expressed both collagen types and that the treatment with EC-I led to an increase in both collagen I and III in all cultured cells. So, we next evaluated by western blot the expression of proteins with a key role in collagen synthesis, P4H and HSP47, on NIH/3T3 cells cultured for 24 h with or without EC-I at 4 mg/ml. Results of densitometric analysis of HSP47 and P4HA1 (the catalytic *α*(I) subunit of P4H [52]) bands normalized versus *β*-actin are shown in Figure 4(b). Data are expressed as mean ± SEM from three independent experiments in duplicate. Representative images of immunoblotting for HSP47 and P4HA1 are also shown. Treatment with EC-I led to a marked and statistically significant increase of HSP47 and P4HA1 levels (*P* < 0.01*vs*. control for both proteins).

### 3.3. Effect of EC-I on Fibroblast Differentiation

**Figure 5:**
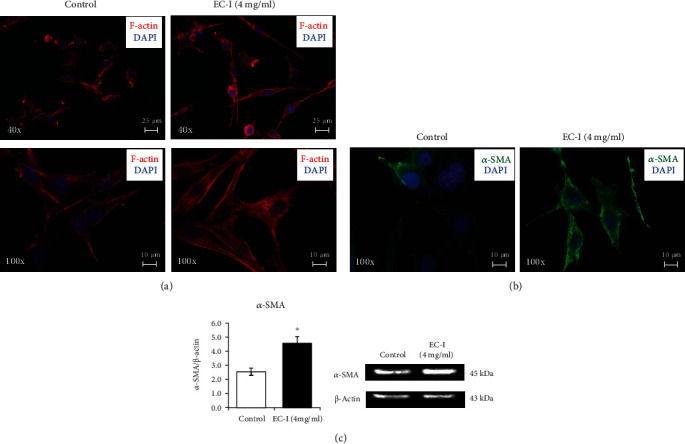
Effect of EC-I treatment on actin cytoskeletal reorganization and *α*-SMA expression level. Immunofluorescence images of NIH 3T3 cells treated for 24 h with EC-I suspension (4 mg/ml) and stained with TRITC-phalloidin (red) to reveal (a) F-actin or (b) anti-*α*-SMA antibody (green). Nuclei were counterstained with DAPI (blue). The images are representative of three independent experiments in duplicate. (c) Western blot analysis of *α*-SMA in NIH 3T3 cells treated with EC-I suspension for 24 h. The densitometric analysis of the bands normalized to *β*-actin reported in the histograms is from three independent experiments in duplicate (mean ± SEM; ∗*P* < 0.05). A representative image of immunoblotting for *α*-SMA is also shown.

As previously reported [53–55], fibroblasts express basal levels of *α*-SMA that increase when they differentiate in myofibroblasts. Of note, the fibroblast myodifferentiation is also associated with a reorganization of *α*-SMA cellular location, being mostly integrated into the cytoskeleton structures. To test whether the increase of collagen content was associated with fibroblast differentiation, we examined the cytoskeletal reorganization and the expression of *α*-SMA, the most used marker of the myofibroblastic phenotype [56, 57]. Consistently, in our experimental conditions, treated fibroblasts expressed higher levels of *α*-SMA, widely located within stress fibers; in contrast, untreated cells showed cytoskeletal microfilaments with a very faint fluorescence staining for *α*-SMA. NIH 3T3 cells incubated with or without EC-I for 24 h were stained with TRITC-phalloidin (red) to reveal F-actin or immunostained for *α*-SMA (green), and the nuclei were counterstained with DAPI (blue) (Figure 5(a)). Exposure to EC-I led to prominent cytoskeletal rearrangement in treated cells, with the formation of well-defined actin in parallel-arranged stress fibers, while control cells showed a rather irregular pattern of F-actin fibers. Moreover, the cells treated with EC-I appeared larger and showed more elongated shape. Immunofluorescence analysis showed, also, an increase of *α*-SMA protein level in treated cells, as evident in the representative images shown in Figure 5(b). Untreated control cells showed a faint green fluorescent, while EC-I-exposed cells displayed an intense *α*-SMA staining. Western blotting analysis also confirmed the increase of *α*-SMA protein in treated cells as quantified through band densitometry. Representative western blots and relative densitometric analyses are shown in Figure 5(c).

**Figure 6:**
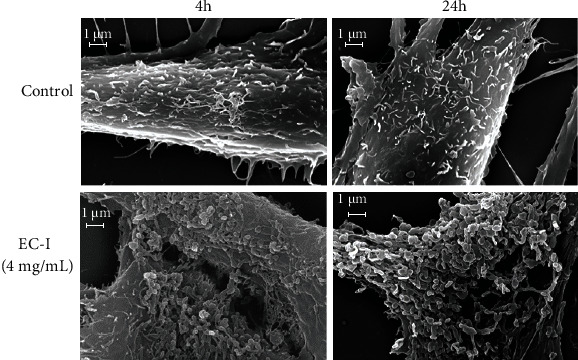
Effect of EC-I treatment on NIH 3T3 morphology. Scanning electron micrograph images of NIH 3T3 untreated (control) or treated with EC-I suspension (4 mg/ml) for 4 and 24 h. (20,000x magnification). Images are representative of two independent experiments in duplicate.

With the aim to further investigate the effect of EC-I on fibroblast morphology, a scanning electron microscopy (SEM) analysis of NIH 3T3 was performed, showing that cells treated with EC-I at 4 mg/ml had a rough surface with numerous microvesicles shedding from the cell membrane. The effect was quite early, being visible already at 4 hours of treatment and increased with incubation time. In fact, the membrane ultrastructures appeared much more numerous at 24 h, almost completely covering the cell body. On the other hand, relatively few protrusions were observed in untreated cells, where the cell membrane seemed smoother, as typical of nonactivated cells. Representative SEM images of untreated (control) or EC-I-treated cells are shown in Figure 6.

## 4. Discussion

In this work, we treated a normal mouse embryo fibroblast cell line, NIH 3T3, with a high concentration micronized type I equine collagen suspension, using a range of concentrations similar to that used in tissue regeneration studies for the preparation of collagen scaffold [48–51]. In our experimental *in vitro* conditions, EC-I had neither a proliferative nor a toxic effect on fibroblasts after 24 h treatment. On the other hand, NIH 3T3 cells showed an increase in collagen levels after the addition of EC-I (4/8 mg/ml). Considering that the effect observed with EC-I at 4 mg/ml was comparable and not significantly different from that exerted by EC-I at 8 mg/ml, we chose the lowest effective dose for subsequent experiments. Type I and type III collagen are the major proteins in dermal ECM [1], covering 80-85% and 10-15% of collagen content, respectively [58], and have considerable roles in healthy human skin during collagen fiber formation. Our results show the ability of EC-I to induce a significant increase in type I and type III collagen levels. Collagen synthesis is regulated by many posttranslational modifications which occur both in intracellular or extracellular space [59]: intracellular events include posttranslational hydroxylation and glycosylation, association of polypeptide chains, and folding of the triple helix; extracellular events include cleavage of the N- and C-propeptides, self-assembly of collagen into fibrils, and cross-linking of the fibrils. Prolyl-4-hydroxylase (P4H), the enzyme which catalyzes proline hydroxylation of procollagen, is essential for collagen maturation and synthesis in fibroblasts [44, 46]; it can be considered as a “rate-limiting enzyme” in collagen production, as its expression levels are associated to the rate of collagen synthesis in both cultured cells and *in vivo* [60]. HSP47 is an endoplasmic reticulum- (ER-) resident molecular chaperone necessary for correct folding of procollagen in mammalian cells, acting in several steps during collagen maturation. HSP47 binds to collagenous (Gly–Xaa–Arg) repeats within triple-helical procollagen in the ER preventing newly formed procollagen chains from aggregating and being degraded in the ER and promoting the stability of the triple-helical region of procollagen, thus accelerating the triple-helix formation of procollagen and aiding collagen secretion [61, 62]. Considering the crucial role of these proteins in collagen biosynthesis, maturation, and secretion, we analyzed the ability of EC-I treatment to influence their expression. Of note, the increase of collagen production induced by EC-I treatment could be associated with an increase in the expression of P4H and HSP47.

We then demonstrated that the EC-I exposure led to an increased expression of *α*-SMA protein, a known marker of myofibroblasts [56, 57], supporting a link between the increase of collagen synthesis and fibroblast differentiation. Myofibroblasts are different from normal fibroblasts in several characteristics, as well as ruffled membranes and a highly active endoplasmic reticulum [63]. Moreover, increased expression of *α*-SMA and vimentin [56] and the formation of stress fibers and specialized adhesion complexes (focal adhesion) are considered all markers of myodifferentiation [63]. Through the interaction between microfilaments and ECM proteins mediated by focal adhesion complexes, myofibroblasts can sense the tension in their surrounding microenvironment and maintain the cellular contractile force via the cytoskeletal protein network. Myofibroblasts respond to this tension by producing ECM proteins, including collagen and remodeling enzymes, such as MMPs [64]. Based on this knowledge, in an attempt to detect possible morphological and cytoskeletal changes in fibroblasts treated with EC-I, we show evidence that the treatment induced a larger and more elongated shape; moreover, it led to cytoskeletal rearrangement with the organization of actin stress fibers and the formation of plasma membrane extensions and blebbing of extracellular vesicles. These morphological changes, along with the increase of *α*-SMA and collagen expression, strongly support that EC-I might be able to induce myodifferentiation. Although the mechanisms underlying the observed effects have yet to be elucidated, some hypotheses can be proposed. As previously reported [65], when the fibroblasts interact with the collagen matrix, isometric tensions develop reaching high levels and the distinct actin stress fibers are promoted in fibroblasts and facilitated by cell-cell contact. In our experimental conditions, as detected by SEM analysis, the water-dissolved EC-I was able to form fibers on which the fibroblasts could adhere, generating tension. Then, the *β*-actin-containing stress fibers integrate the *α*-SMA present in the fibroblast cytosol and the G-actin involved in the polymerization releases the transcription factor MKL1 which, after nuclear translocation, leads to the increase of the *α*-SMA gene expression. These changes result in the formation of the cytoplasmic actin microfilament system containing *α*-SMA, a typical feature of myofibroblasts [65]. In addition, the physical interaction between fibroblasts and collagen could be also mediated by integrin and/or discoidin domain receptors that serve as mechanolinks, leading to the activation of the important signaling pathways, involved in cell activation, proliferation, MMP induction, and collagen neosynthesis [65–71].

As far as we know, this is the first study on the cellular effects of a commercially available formulation of heterologous collagen aqueous solution, used for almost 30 years in the healing of skin ulcers, bedsores, and postoperative wounds, with no side effects. Currently, this product is being used by specialists also in anti-skin aging medicine, as an adjuvant in skin biorevitalization favouring the regeneration of the connective tissue of the dermis, creating the optimal conditions for the physiological neosynthesis of collagen. In this regard, it is important to point out that, despite recombinant human collagen is emerging as a potential production method, the use of this type of collagen is limited due to a number of disadvantages such as low yield, high cost, and issues compromising the stable formation of bioactive and biofunctional collagens. For these reasons, extracted animal collagen has remained the standard for use in both research and clinical fields [28, 72, 73]. Finally, even though approaches to preventing cellular aging should be realized in young cells rather than senescent ones, it could be of interest to verify in the near future if the effects observed in our experimental conditions are reproducible on matured or aged human fibroblasts.

## 5. Conclusions

In conclusion, our findings show the ability of a type I equine collagen aqueous suspension, largely used for skin rejuvenation, to induce collagen synthesis and myodifferentiation in a mouse embryo fibroblast cell line. Even if further research is required to identify the molecular and biochemical mechanisms underlying the observed effects, our results also support the suitability of the *in vitro* cell model to verify if collagen peptides are absorbed by the cells or act as stimulatory signals at the membrane level.

## Data Availability

The data used to support the findings of this study are available from the corresponding author upon request.
